# 

**Published:** 1907-08-10

**Authors:** 


THE HOSPITAL *August* 10, 1907.

*Name* ..........

*Address* ..........

..........

This Coupon must accompany manuscript or contributions intended for The Hospital.

